# Supplementary material for: Increase in blood pressure precedes distress behavior in nursing home residents with dementia
Source: PLoS One. 2024 Apr 30;19(4):e0298281. doi: 10.1371/journal.pone.0298281 (PMC11060555; doi:10.1371/journal.pone.0298281)
Supplement: S1 Fig — (DOCX) [file pone.0298281.s001.docx]

S1 Figure. Distress Behavior in Dementia Incident Cohort Construction; DBD = Distress Behavior in Dementia; CFS = Cognitive Function Scale

Removing individuals with CFS = 0 and without dementia

N=14

Individuals included in either 2019 or 2020 but not both

N=256

DBD incidents (individuals) in 2019

N=4,204 (557)

DBD incidents (individuals)

N=9,787 (993)

DBD incidents (individuals) in those with CFS > 0 or dementia

N=9,772 (979)

DBD incidents (individuals) in 2020

N=5,583 (692)

DBD incidents (individuals) in those with blood pressures recorded on the DBD incident day and on a comparison day within 7 days before DBD incident

N=3,152 (707)
