# Supplementary material for: Increase in blood pressure precedes distress behavior in nursing home residents with dementia
Source: PLoS One. 2024 Apr 30;19(4):e0298281. doi: 10.1371/journal.pone.0298281 (PMC11060555; doi:10.1371/journal.pone.0298281)
Supplement: S2 Fig — (DOCX) [file pone.0298281.s002.docx]

S2 Figure. Comparison Cohort Construction; DBD = Distress Behavior in Dementia; CFS = Cognitive Function Scale

Removing individuals with CFS = 0 and without dementia

N=2,736

Blood pressure values (individuals) -- in those without DBD incidents -- recorded on the same day as the DBD incident in the Incident cohort

N=191,373 (6,376)

Blood pressure values (individuals) -- in those without DBD incidents -- and with CFS > 0 or dementia

N=80,979 (3,625)

Blood pressure values (individuals) -- in those without DBD incidents -- recorded on the same day as the DBD incident in the Incident cohort and on a comparison day within 7 days before DBD incident

N=8,288 (2,328)

Removing records with missing values (individuals)

N=3,070 (15)
